# Liver transplantation in Jehovah’s witnesses: 13 consecutive cases at a single institution

**DOI:** 10.1186/s12871-020-0945-x

**Published:** 2020-01-30

**Authors:** Diego Costanzo, Maria Bindi, Davide Ghinolfi, Massimo Esposito, Francesco Corradi, Francesco Forfori, Paolo De Simone, Andrea De Gasperi, Gianni Biancofiore

**Affiliations:** 1 Transplant Anesthesia and Critical Care Unit, University School of Medicine, Azienda Ospedaliera-Universitaria Pisana, Pisa, Italy; 2 Liver Transplant Surgery Unit, University School of Medicine, Azienda Ospedaliera-Universitaria Pisana, Pisa, Italy; 3 Anesthesia and Critical Care Unit, Ospedale Niguarda Ca’ Granda, Milan, Italy

**Keywords:** Liver transplantation, Jehovah witnesses, Bloodless medicine and surgery programs; strategies, Transfusion-alternative

## Abstract

**Background:**

Jehovah’s Witnesses represent a tremendous clinical challenge when indicated to liver transplantation because they refuse blood transfusion on religious grounds and the procedure is historically associated with potential massive peri-operative blood loss. We herein describe a peri-operative management pathway with strategies toward a transfusion-free environment with the aim not only of offering liver transplant to selected Jehovah’s Witnesses patients but also, ultimately, of translating this practice to all general surgical procedures.

**Methods:**

This is a retrospective review of prospective medical records of JW patients who underwent LT at our Institution. The peri-operative multimodal strategy to liver transplantation in Jehovah’s Witnesses includes a pre-operative red cell mass optimization package and the intra-operative use of normovolemic haemodilution, veno-venous bypass and low central venous pressure.

**Results:**

In a 9-year period, 13 Jehovah’s Witness patients received liver transplantation at our centre representing the largest liver transplant program from deceased donors in Jehovah’s Witnesses patients reported so far. No patient received blood bank products but 3 had fibrinogen concentrate and one tranexamic acid to correct ongoing hyper-fibrinolysis. There were 4 cases of acute kidney injury (one required extracorporeal renal replacement treatment) and one patient needed vasoactive medications to support blood pressure for the first 2 postoperative days. Two patients underwent re-laparotomy. Finally, of the 13 recipients, 12 were alive at the 1 year follow-up interview and 1 died due to septic complications.

**Conclusions:**

Our experience confirms that liver transplantation in selected Jehovah’s Witnesses patients can be feasible and safe provided that it is carried out at a very experienced centre and according to a multidisciplinary approach.

## Background

The religion of Jehovah’s Witnesses (JW) was founded in the nineteenth century and its community counts about 250.000 members in Italy (https://www.jw.org/it/testimoni-di-geova/nel-mondo/IT). Liver transplantation (LT) is the only therapeutic option for patients with end-stage liver disease (ESLD) or acute liver failure and its outcomes markedly improved over the last decades [1]. Since the risk of life-threatening peri-operative bleeding in LT remains considerable and JW refuse blood transfusion on religious grounds, they always represented a tremendous clinical challenge and generated ethical and legal concerns when indicated to this procedure [2]. At the University of Pisa, Azienda Ospedaliera-Universitaria Pisana, we developed a peri-operative management pathway with strategies toward a transfusion-free environment with the aim not only of offering LT to selected JW patients but also, ultimately, of translating this practice to all general surgical procedures. Since there are so few LT centers willing to perform LT on JW patients and literature detailing their peri-operative care and outcomes is poor, in this report we describe our experience of LT in JW and analyze feasibility and safety and evaluate the optimal management.

## Methods

This is a retrospective review of prospective medical records of JW patients who underwent LT at our Institution from 2007 to 2016. The study was approved by the local ethical committee, Comitato Etico Area Vasta Nord Ovest, Pisa (Nr 1552; 03/08/2018).

### Preoperative management

JW patients were evaluated as per our standard Institutional clinical protocol. For this particular class of patients, two or more indicators of severe portal hypertension (platelets < 50 × 10^3^ μL, grade 3 esophageal varices, ascites, previous variceal hemorrhage and hepatic encephalopathy) were considered contraindications for listing [3] as well as INR > 2.5 and stage 2 kidney injury according to the AKIN classification. Patients were deactivated from the list if, due to worsening of their clinical condition, they fell outside these criteria. Patients with previous upper abdominal surgery and UNOS status 1 and 2 were also excluded. Once indicated to LT and provided that involved senior surgeons and anesthesiologists agreed to perform the procedure, patients were requested to attend two interviews: one in presence of the next of kin and a JW elder and one alone with our centre’s clinical leadership. All patients were required to sign a consent form that reflected their wishes formally refusing the use of blood products even in case of life-threatening anemia and stating the accepted therapies. In fact, since acceptance of blood fractions by JW depends on patient’s free will apart from church doctrine [4], there are some therapies that all JW refuse (whole homologous blood and its main fractions: plasma, red blood cells, white blood cells, platelets, and preoperative storage of autologous blood for later use during surgery) whereas there are some other treatments that most, but not all, Witnesses accept (perioperative blood salvage; products derived from the main components of blood, such as albumin, clotting factors, antithrombin III, synthetic hemoglobin, autotransplantation of stem cells, transplant of solid organs, normovolemic hemodilution). Finally, some other therapies are accepted by all Witnesses: plasma substitutes, which are not derived from blood, erythropoietin and hemopoietic agents obtained from genetic recombination [4]. Therefore, all of the JW candidate to LT received detailed and written information about each of the available therapies. Following admission to the waiting list, patients showing an Hemoglobin (Hb) level < 12 mg/dL underwent hematological consultation in the aim to optimize their red cell mass. We developed a preoperative protocol for JW candidates to LT which includes supplementation with iron or vitamin (vitamin B12, folic acid) and subcutaneous recombinant erythropoietin (40.000 IU weekly). The hematological package inserts also monthly laboratory checks and interviews in order to adjust treatments. Transplant candidates with Hb > 12 mg/dL were monitored with regular laboratory checks and, if necessary, they were included in the optimization protocol.

### Intra-operative management

Standard anesthetic management was used as already reported [5]. During hepatectomy and the anhepatic phase (the time from the physical removal of the native liver until the revascularization of the graft), patients were managed by the attending anesthetist aiming at a low central venous pressure (CVP), ideally ≤5 mmHg, by restrictive volume infusion. Intra-operative cell salvage (ICS) and acute normovolemic hemodilution (ANH) were adopted whenever feasible. The ICS (Cell Saver 4, Haemonetics Corporation, Braintree, MA, USA; Dideco Electa; Sorin Group, Miradola, Italy) was used in all procedures and its circuit was maintained in continuity with patient’s circulation. The volume of red cells returned to patients from ICS was used as an estimation of intra-operative bleeding [6]. For the purposes of ANH, we contraindicated it in patients with Hematocrit (Hct) < 35%, platelets count < 100.000 and INR > 1.5. In case of ANH, blood was removed from a central line by gravity and drained to a citrate-phosphate-dextrose (CPD) bag after anesthesia induction with the patient’s intravascular volume being maintained by infusion of crystalloid solutions. The CPD tubing remained connected to the patient at all times conforming to the patients’ religious beliefs. Hypothermia was limited by the use of forced air warmer blankets and intravenous fluids warmers. All LTs were performed using the conventional surgical technique with vena cava replacement and veno-venous bypass. Coagulation profiles were tracked using rotational thromboelastography and patients’ approved component therapies were administered accordingly. Finally, experienced surgeons and anesthetists were in charge of all cases.

### Post-operative management

After surgery, all patients remained under observation in the ICU and then transferred to the regular ward. Optimal basic conditions for coagulation was ensured and antifibrinolytic treatment was initiated early if needed. In case of sub-optimal oxygenation, general anesthesia, intubation and hyperoxic ventilation were considered early. Post-operative cell salvage of drainage blood was implemented in case of massive production and surgical re-intervention was considered immediately in case of worsening anemia. Laboratory tests were reduced to a minimum and, if necessary, performed with low volume blood sampling systems. Postoperativelly, erythropoiesis was stimulated when needed following the same criteria and protocol used for pre-OLT preparation.

### Statistical analysis

Results are reported as median [IQR]. One-way analysis of variance (ANOVA) with Tuckey’s correction for multiple comparisons was used for statistical analysis and the significance was set at *p* < 0.05. Data analysis was performed using GraphPad Prism (version 7.00 for Windows, GraphPad Software, La Jolla, CA, USA).

## Results

Since 2007, 16 JW patients were considered for LT at our Institution: 3 of them showed more that 2 indicators of severe portal hypertension and were not admitted to the transplant whereas the other 13 were listed and received a graft from a deceased donor. Age at the time of LT was 51 [44.5–57.5] years with a BMI of 23.7 [20.8–26.9] kg/m2. MELD score, the scoring system for assessing the severity of chronic liver disease [7], was 15 [12–22] and the diseases indicating LT were post infection liver cirrhosis (*n* = 8, 61.5%) which was associated with hepatocellular carcinoma in the 75% of cases (*n* = 6), alcoholic cirrhosis (*n* = 2, 15.4%), primary biliary cirrhosis (*n* = 1, 7.7%), alpha-1 antitrypsin deficiency (n = 1, 7.7%) and hemochromatosis (*n* = 1, 7.7%), (Table 1). One patient was thalassemic and another had a previous transjugular intrahepatic portosystemic shunt due to repeated variceal bleeding. Waiting time before LT was 189 [58.5–390.5] days. Graft’s cold and warm ischemia times were 460 [425.5 to 528.5] and 74 [68–86.5] minutes respectively and total surgery duration was 435 [420 to 450] minutes. Patients Hct at the time of listing was 38.5 [35.1–42.3] % with an hemoglobin level of 12.8 [11.8–15.4] mg/dL. Two patients needed red cell mass optimization and the targeted Hct was achieved within 2 months. All of the transplanted patients agreed to receive AHN, ICS and, in case of need, coagulation factors concentrates and fibrinogen concentrate. A comprehensive report of patients hematologic profile is reported in Table 2 whereas Table 3 shows individual data. ANH was performed in 6 patients with 3 [2.7–3] retrieved Units. The volume of red cells returned to patients from ICS was 400 [217–720] mL. No patient received blood bank products, 3 had fibrinogen concentrate 2 g and one also needed 2 g tranexamic acid to correct ongoing hyper-fibrinolysis. The amount of fluids infused during surgery was 3560 [3425–4300] mL, all balanced crystalloid solutions. ICU and hospital length of stay were 4 [3–5] and 18 [11–31] days respectively. There were 4 cases of acute kidney injury (one required extracorporeal renal replacement treatment). Two patients received nor-epinephrine at reperfusion (Table 1), in one of them it was continued for the first 2 postoperative days (peak dose 2.5 mcg/kg/min) to support blood pressure. Two patients underwent re-laparotomy: one on post-operative day (POD) #4 for a large abdominal hematoma wash-out with no detectable source of bleeding and another on POD #14 due to duodenal perforation. In both cases patients did not show signs of coagulopathy and did not receive transfusions. One patient developed an urinary tract infection on POD #5 due to *Escherichia coli*. Finally, of the 13 JW recipients, 12 (92.3%) were alive at the 1 year follow-up interview: 1 patient died 11 months from LT due to ischemic-type biliary lesions (ITBL)-related septic complications.

**Table 1:** Patients demographic and perioperative data

year of LT	Pre-LT disease	MELD score	Surgery duration (minutes)	Cold/Warm ischemia (minutes)	In-hospital complications	Post LT ICU LoS (days)	Hospital LoS (days)	1 year outcome
2007	HCC-HCV	12	400	477/78		4	18	Alive
2008	HBV	22	450	512/60		5	20	Alive
2009	HBV-HCV	13	400	495/70	Bleeding POD #4, AKI	13	28	Alive
2009	HCC-HCV-HBV	24	480	437/73		3	18	Alive
2009	HCC-HCV	15	450	640/84		2	13	Alive
2011	Primary biliary cirrhosis	25	420	450/60		3	12	Dead
2011	Hemochromatosis	22	490	613/113	• Intraoperative reperfusion syndrome (nor-epinepinephrine 2.5 mcg/kg/min) • post-operative AKI	5	15	Alive
2011	alpha-1 antitrypsin deficiency	18	420	460/90	Abdominal perforation POD#14; AKI, CRRT	4	55	Alive
2011	alcoholic	22	420	423/66		2	13	Alive
2013	alcoholic	11	435	412/88		4	14	Alive
2013	HCC-HCV	8	450	400/70	• Intraoperative reperfusion syndrome (nor-epinepinephrine 2.0 mcg/kg/min) • Post-operative AKI	4	20	Alive
2014	HCC-HCV	15	445	428/74	Urinary tract infection POD #5	3	18	Alive
2016	HCC-HCV	12	425	545/85		3	12	Alive

*HCC* hepatic cell carcinoma, *HBV* hepatitis B virus, *HCV* hepatitis C virus, *POD* post operative day, *LoS* length of stay

**Table 2:** Peri-operative hematologic profile (all patienns)

	Hct	ANOVA
Time of listing, %	38.5 [35.1 to 42.3]	
Start of Surgery, %	40.2 [35.8 to 42.8]	
End of Surgery, %	33.2 [29.7 to 34.7]	*P* < 0.01 Vs Start of Surgery
Discharge home, %	33.1 [29.4 to 35.6]	*P* < 0.01 Vs Start of Surgery
	Hbcenter	ANOVAcenter
Time of listing, g/dL	12.8 [11.8 to 15.4]	
Start of Surgery, g/dL	14.0 [13.3 to 15.5]	
End of Surgery, g/dL	10.4 [9.2 to 11.7]	*P* < 0.05 Vs Time of listing *P* < 0.0001 Vs Start of Surgery
Discharge home, g/dL	13.1 [11.3 to 13.5]	*P* < 0.01 Vs Start of Surgery *P* < 0.01 Vs End of Surgery
	INRcenter	ANOVAcenter
Time of listing	1.5 [12 to 1.8]	
Start of Surgery	1.7 [1.2 to 1.9]	
End of Surgery	2.2 [1.9 to 2.5]	*P* < 0.01 Vs Time of listing *P* < 0.01 Vs Start of Surgery
Discharge home	1.2 [1 to 1.5]	*P* < 0.0001 Vs End of Surgery
	PLTcenter	ANOVAcenter
Time of listing, 10^3^ μL	100 [51 to 208]	
Start of Surgery, 10^3^ μL	100 [55.5 to 201]	
End of Surgery, 10^3^ μL	44 [39 to 109.5]	*P* < 0.05 Vs Time of listing *P* < 0.001 Vs Start of Surgery
Discharge home, 10^3^ μL	120 [98.5 to 172]	*P* < 0.0001 Vs End of Surgery

Data are median and [IQR]; *Hct* hematochrit, *Hb* hemoglobin, *INR* international normalize ratio, *PLT* platelets;

**Table 3:** Peri-operative hematologic profile (individual data)

Patient #	Hct (%)				Hb (g/dL)				INR				PLT (10^3^ μL)				ANH Units	ICS mL
	ToL	SoS	EoS	Dis	ToL	SoS	EoS	Dis	ToL	SoS	EoS	Dis	ToL	SoS	EoS	Dis		
1	42.0	42.5	32.9	32.6	15.3	15.8	8.7	11.2	1.5	1.4	1.8	1.2	100	100	44	95	3	1250
2	41.5	40.2	35.3	33.1	15.1	15.8	12.8	13.2	1.6	1.7	1.4	1.1	76	89	42	91	=	234
3	49.1	48.2	28.3	34.8	17.1	16.4	9.4	13.5	1.2	1.2	1.9	0.9	207	195	122	189	3	400
4	38.5	39.8	33.5	35.5	12.7	12.9	11.5	13.1	2.0	2.0	2.6	1.5	52	51	40	105	=	110
5	42.6	40.4	33.2	29.9	15.5	15.2	9.7	11.4	1.7	1.7	2.1	1	49	46	29	97	=	980
6	38.6	33.6	29.0	30.0	12.8	13.2	9.1	11.9	2.0	2.1	2.6	1.2	50	89	61	100	=	460
7	37.1	33.9	30.4	28.5	12.7	14.0	10.4	12.7	1.2	1.2	2.0	1.1	215	220	134	155	=	400
8	30.2	40.1	33.5	25.9	9.8	13.8 (R)	11.4	10.5	1.5	1.5	1.9	1.3	127	130	97	120	2	390
9	34.2	33.4	30.6	39.2	11.5	13.2	10.8	13.5	1.8	2.1	2.3	1	200	199	44	132	=	200
10	36.1	40.4	36.7	36.7	12.1	14.9	12.0	13.1	1.9	1.8	2.2	1.3	83	60	38	141	=	100
11	27.1	37.8	34.1	35.8	9.6	13.5(R)	10.1	14.1	0.9	1.0	2.5	1.8	210	370	207	205	3	460
12	47.2	44.1	20.9	29.0	15.6	13.4	7.3	10.4	1.1	1.2	2.6	1.5	248	203	95	200	3	1500
13	37.5	43.2	37.4	35.5	13.5	14.8	12.6	13.8	1.4	1.8	2.3	1.5	46	44	31	102	3	300

*Hct* hematochrit, *Hb* hemoglobin, *INR* international normalize ratio, *PLT* platelets, *ANH* acute hemodilution normovolemic, *ICS* Intraoperative cell salvage, *ToL* time of listing, *SoS* start of surgery, *EoS* end of surgery, *Dis* discharge home, (*R*) after red cell mass optimization protocol

## Discussion

Two key factors led us in deciding to offer LT to JW patients. The first was our large experience in the procedure. In fact, we transplanted our first JW patient in 2007, 11 years and 930 procedures from the start of our LT program in 1996. The second was the rate of bloodless LT characterizing our activity in the years prior to that decision [5]. In our experience, a careful selection of recipients was a key-player as we decided to admit JW patients to the pre-LT screening only if they did not show indicators of severe portal hypertension since bleeding in LT is predominantly linked to portal hypertension rather than primary coagulopathy [8–12]. Thus, early referral and careful timing for listing is highly desirable in these patients. Predicting cases requiring peri-operative transfusions is very desirable, particularly in JW patients, but it remains a very difficult task in LT [8, 11, 12]. In fact, despite several investigations have attempted to identify preoperative predictors of blood transfusion, their value remain inconsistent and weak [2, 8–14]. In recent years, models to predict blood utilization with preoperative variables have been proposed [11, 14, 15]. However, their limitations and differences in the results highlight significant concerns about their generalizability and recognize that it may be very difficult to develop a single, reliable, and universally applicable model to predict transfusion requirements for patients undergoing LT. In our series, the use of techniques that minimize blood loss played a major role. The adoption of a fluid restriction policy and low CVP is important as it results helpful in decreasing blood transfusion requirements during LT [8, 16]. In fact, liberal volume loading in cirrhotic patients tends to pool in the splanchnic circulation with minimal improvement in cardiac preload or output but increased risk of surgical bleeding because of congestion of the portal circulation [9, 10, 15]. Moreover, dilution of clotting factors and clot disturbance can result, particularly if colloids are used [2, 13]. Further, lowering the pressure in the central veins can help in minimizing blood loss also because it may augment venous drainage from the liver, encouraging flow of blood away from the surgical field [9–12]. Other intra-operative blood conservation strategies can be important to achieve transfusion-free surgery. In our JW patients we performed ANH but only in selected patients in order not to excessively dilute clotting factors, including PLT. Finally, in the view of a multimodal strategy aimed at reducing blood loss [9], we used in all cases the veno-venous bypass to minimize the impact of mesenteric congestion and abdominal bleeding during portal and caval cross-clamping [17]. In case of peri-operative coagulopathy, since JW will not accept fresh frozen plasma or platelets, the use of coagulation factor concentrates and/or tranexamic acid guided by rotational thromboelastometry (ROTEM®) or thrombelastography (TEG™) [8–11] should be considered. Therefore, the use of fibrinogen concentrate (which can support fibrin clotting without transfusion of PLT), clotting factors concentrates and tranexamic acid should be explained and proposed to these patients in the consideration that acceptance of blood fractions by JW depends on patient’s free will, apart from church doctrine [4, 13]. Regarding the use of recombinant erythropoietin to optimize red cell mass, it has a number of potential benefits. The most obvious is that patients will start LT with a higher blood Hb level that, besides enabling use of ANH [9, 10], correlates with peri-operative low or no use of red blood cells transfusion [2, 18]. Since erythropoietin’s use was associated to a possible risk of thromboembolism [18], we performed regular clinical and laboratory monitoring of LT candidates on erythropoietin treatment also in consideration that the waiting time for a suitable deceased donor is unpredictable. The use of ICS in patients with hepatocellular carcinoma could be discussed because of the potential risk of infusing malignant cells into patients. To date, 4 studies have evaluated the oncological safety of using ICS in LT. One concluded that the device is effective in removing malignant cells from the aspirate, except in cases of tumor rupture whereas the other 3, evaluating clinical outcomes such as mortality and recurrence, did not demonstrate negative effects associated with the use of ICS [19]. On the contrary, the use of cell salvage during LT for hepatocellular carcinoma has been found to reduce the exposure to allogeneic blood and to be cost-effective [20]. In summary, also according to the most recent guidelines [21], despite theoretical risks and benefits, there is no conclusive evidence that ICS can induce metastases or affect cancer prognosis. The theoretical risk of inducing metastatic spread (unproven) is offset by reduced allogeneic transfusion and immunomodulation, which is proven [21]. During the last decade, blood product requirements in LT patients have significantly decreased in most centres. This improvement was related to different factors including better surgical techniques, LT indication and liver graft preservation techniques [8–11]. Also, experience of the surgical and anesthesiological team is important. In particular, surgical experience and skill during hepatic dissection and meticulous hemostasis has long been recognized as meaningful in determining the amount of intraoperative blood loss [22].

However, experience is difficult to quantify and many unforeseen intraoperative events with the potential occurrence of technical difficulties impart complex changes predisposing to extensive bleeding. Furthermore¸ there is evidence that transfusional requirements can be reduced if the anesthesia team followed protocols including goal-directed transfusion practices [23]. However, comparison of intraoperative transfusion requirements from different transplant centers may be inherently biased by an inability to account for differences in transfusion triggers and clinical practices. Consequently, the predictive models developed in one institution may hardly, if ever, be applicable in others.

Few other cases of LT in JW have been reported so far and, since the first one ever in 1994 [24], our series is, to the best of our knowledge, the second most numerous overall and the largest from deceased donors. In fact, Jabbour and colleagues from University of Southern California reported in 2005 the results of 27 consecutive LT in JW patients, 19 from living and only 8 from deceased donors whereas Detry and colleagues reported 6 cases from Liege in Belgium [6, 25]. Other smaller case series are available from different countries [26–28].

It is undeniable that a rather diffused concern exists about offering LT to JW patients. In fact, the acceptance of solid organ transplantation and contemporary refusal of transfusion are hardly understandable for non-JW. However, it is important to remember that, as far as organ transplants are concerned, each Witness is called on to decide personally whether to accept them or not, with the proviso that no part of the transplantation process may include the use of blood or its main fractions [4, 13]. Therefore, each JW patient should be interviewed individually excluding external pressure and the use of every available peri-operative option aimed at a blood sparing management, including the use of factors and fibrinogen concentrates, should be carefully discussed and clarified. Since our patients have been transplanted over a period of 10 years, it could be argued that peri-operative management policies could have changed during this period with a possible effect of time on results. However, no substantial changes in clinical care of recipients were made during the considered period and there was also consistency in anesthesiology and perioperative treatments.

## Conclusion

We herein presented the results of the largest LT program from deceased donors in JW patients that has been reported so far. Our experience shows that the risk-to-benefits ratio of LT can be maintained in selected adult JW patients provided that it is carried out at a very experienced centre and according to a multidisciplinary approach. We reported our experience in the aim to offer a template for a broader concept of transfusion-free surgery principles rather than as a focus on a technical achievement in such a complicated clinical setting. Anesthesiologists, as users of blood products, are called to lead a continuous re-evaluation process aimed at defining the most proficient approach to bloodless surgery and at contributing to the debate about benchmarking in difficult clinical scenarios.

## Data Availability

The datasets used and/or analysed during the current study are available from the corresponding author on reasonable request.

## Abbreviations

AHN: Acute normovolemic hemodilution
CPD: Citrate-phosphate-dextrose
CVP: Central venous pressure
ESLD: End-stage liver disease
Hb: Hemoglobin
Ht: Hematocrit
ICS: Intra-operative cell salvage
ICU: Intensive care unit
JW: Jehovah’s Witnesses
LT: Liver transplantation
